# The Tobacco Endgame—A New Paradigm for Smoking Cessation in Cancer Clinics

**DOI:** 10.3390/curroncol29090497

**Published:** 2022-09-01

**Authors:** Emily Stone, Christine Paul

**Affiliations:** 1 Department of Thoracic Medicine and Lung Transplantation, St Vincent’s Hospital Sydney, Darlinghurst 2010, Australia; 2 School of Clinical Medicine, University of New South Wales, Sydney 2052, Australia; 3 School of Public Health, University of Sydney, Camperdown 2006, Australia; 4 School of Medicine and Public Health, University of Newcastle, Callaghan 2308, Australia

**Keywords:** cancer, lung cancer, smoking cessation, tobacco endgame

## Abstract

Smoking cessation represents an untapped resource for cancer therapy. Many people who smoke and have cancer (tobacco-related or otherwise) struggle to quit and as a result, jeopardise response to treatment, recovery after surgery and long-term survival. Many health care practitioners working in cancer medicine feel undertrained, unprepared and unsupported to provide effective smoking cessation therapy. Many institutions and healthcare systems do provide smoking cessation programs, guidelines and referral pathways for cancer patients, but these may be unevenly applied. The growing body of evidence, from both retrospective and prospective clinical studies, confirms the benefit of smoking cessation and will provide much needed evidence for the best and most effective interventions in cancer clinics. In addition to reducing demand, helping cancer patients quit and treating addiction, a firm commitment to developing smoke free societies may transform cancer medicine in the future. While the Framework Convention for Tobacco Control (FCTC) has dominated global tobacco control for the last two decades, many jurisdictions are starting to develop plans to make their communities tobacco free, to introduce the tobacco endgame. Characterised by downward pressure on tobacco supply, limited sales, limited access and denormalization of smoking, these policies may radically change the milieu in which people with cancer receive treatment, in which health care practitioners refine skills and which may ultimately foster dramatic improvements in cancer outcomes.

## 1. Introduction

Despite recent advances in tobacco control in many countries around the world, the risks of tobacco exposure for people with cancer remain high. People with cancer often have higher smoking rates than the general population. Those who smoke understand the risks to varying degrees but usually want to quit [1]. Smoking cessation treatments, a crucial part of cancer therapy, can vary widely. Many governments have concentrated on reducing demand for tobacco with quit campaigns, smoking bans and taxation. The Framework Convention for Tobacco Control [2] provides international policy benchmarks for tobacco control. The World Health Organisation (WHO) MPOWER measures [3] provide guidance for policy makers in designing tobacco control and smoking cessation programs, emphasising assistance to quit, warnings about the dangers of tobacco, smoking bans and raising taxation. Smoking cessation guidelines provide guidance on pharmacotherapeutic steps [4] as well as behavioural interventions [5]. Advice and recommendations for people with cancer may relate more to specific tumour streams or screening [6,7,8] than to comprehensive cancer treatment programs. Novel smoke free or tobacco endgame policies may foster new treatment environments for people with cancer by diminishing the influence of the tobacco industry. This study will provide a narrative review of the evidence for tobacco endgame strategies that reinforce optimal treatment for people with cancer, either now or in the future. Specific contention between conventional pharmacotherapy and electronic nicotine delivery devices in the treatment of people with cancer remains outside the scope of this paper.

## 2. Smoking in People Attending Cancer Clinics

Although the link between tobacco smoke exposure and many cancers has been evident for many years, many people with cancer continue to smoke. Data from studies in tobacco-related cancers show high rates of smoking with many patients relatively unaware of the risks [8,9,10,11,12]. A survey of over 1300 cancer patients across Australia recorded a smoking rate of 22% in people with lung cancer, especially in younger patients (<65 years) and in lower socioeconomic groups [9]. In a study of over 100 urology outpatients, 64% were current or former smokers; overall, the respondents recognised the smoking-related risk for other diseases (lung cancer, head and neck cancer (HNC)) more than for urological conditions [10]. In a Finnish survey of nearly 400 patients with bladder cancer, just over half recognised smoking as a risk factor, and overall, patients knew more about it as a risk factor for other conditions including lung cancer and vascular disease [11]. Smoking may confer poorer outcomes on people with cancer. A 2019 meta-analysis reviewed data from over 6000 patients with HNC after radiotherapy and demonstrated poorer outcomes in those who continued to smoke with nearly twice the mortality, higher locoregional recurrence and greater risk of radiation-induced toxicity [12]. A 2022 Taiwanese registry study of nearly 19,000 people with colorectal cancer demonstrated higher mortality in current or former smokers, particularly in those who smoked for more than 10 years and more than 10 cigarettes per day [13].

## 3. Health Care Practitioners’ Perspectives on Smoking Cessation Care in Cancer Clinics

Health care practitioners (HCPs) who work in cancer clinics commonly ask about smoking, with a review finding that more than 75% of oncology cancer clinicians assessed tobacco use during an intake visit [14]. However, HCPs and wider health systems may underestimate the importance of smoking cessation treatment for these patients and may not prioritise cessation care as part of clinical practice. Common misunderstandings held by HCPs may include beliefs that patients who smoke do not want to quit, that quitting in the context of incurable disease is futile and that “it is not my job to address smoking cessation” [15]. From a USA tobacco use survey of nearly 10,000 current smokers, nearly one-third of those with cancer did not receive tobacco cessation advice, and fewer than one-fifth had used approved pharmacotherapy in the preceding 12 months [16]. Analysis of data from a US-based tobacco use survey showed gaps in the provision of smoking cessation treatment in cancer care [16]. In the cohort of people with a history of cancer who were current smokers, most (73%) had received smoking cessation advice, but a minority (18%) had used pharmacotherapy in the previous year. Smoking cessation may be hard to implement in cancer clinics. In the first 18 months of a tobacco treatment program for over 26,000 patients with cancer, only 17% of current smokers accepted assistance [17]. This appears to reflect primary care settings where more patients receive advice on quitting than pharmacotherapy [18]. A cancer diagnosis, however, does increase intention to quit [19] (the “teachable moment”) and increases the likelihood of quitting even though smoking prevalence remains high [20]. Quitting after a cancer diagnosis can significantly improve outcomes. A recent meta-analysis investigating the prognostic effects of smoking cessation around the time of lung cancer diagnosis found, in 21 studies involving over 10,000 patients, that quitting was associated with improved survival for both NSCLC and SCLC, both early stage and advanced [21].

## 4. Tobacco Treatment in Cancer Clinics and Hospital Settings

Interventions and approaches to smoking cessation in cancer clinics vary between different jurisdictions and settings. A diagnosis of cancer may motivate patients towards tobacco treatment, particularly with smoking-related malignancies such as HNC [7]. However, for those diagnosed with a smoking-related cancer, there can be reluctance to engage with a smoking cessation service, which is perhaps due to perceived guilt or shame [22]. A 2016 systematic review of smoking cessation treatment in head and neck cancer (HNC) found just three relevant studies (that reported a measure for smoking cessation), comprising just under 550 patients [23]. All three had control groups, and two of the three studies were randomised (RCT). Interventions included pharmacological and/or behavioural strategies, and only one of the three studies (RCT testing cognitive behavioural therapy with pharmacotherapy) demonstrated higher quit rates with intervention. In another prospective study of just over 70 current smokers on curative cancer therapy, smoking cessation with a motivational interview and (if appropriate) pharmacotherapy demonstrated prolonged quit rates in 21% [24]. Success was associated with a greater initial readiness to stop smoking but not (in this small study) with the use of pharmacotherapy. In a German otorhinolaryngology clinic, fewer than half of the patients surveyed had been offered smoking cessation during treatment for HNC or other smoking-related conditions [6]. Analysis of data from 38 US-based cancer centres found that 28% of smokers received at least one type of cessation treatment and that automated methods to identify and contact them worked better than in-person methods (55% vs. 18% contacted) [25]. Of those who received cessation treatment, the 6-month abstinence rate was higher than background at 18%.

Many HCPs working in cancer medicine do not feel competent in the delivery of smoking cessation therapy. An Australian survey of several hundred oncologists found that although most asked patients about a smoking history, fewer than 20% regularly discussed medication options, and fewer than 5% actively provided smoking cessation therapy [26]. Most (over 95%) preferred that other HCWs provide smoking cessation therapy to their patients.

Hospitals may struggle to embed smoking cessation into routine care, both generally and in cancer clinics. In a systematic review of 63 studies investigating the implementation of smoking cessation in hospital settings, the focus on staff training outweighed efforts in planning, allocation of resources and delegation of tasks [27]. In a qualitative study with hospital staff, interviews in rural, regional and metropolitan hospitals identified concerns about lack of time to deliver smoking cessation therapy, the need for clearer policies, for better training and for consistent messaging [28].

Several studies have investigated specific components of smoking cessation programs for cancer clinics with further, randomised trials underway. Cancer patients may need information about the harmful effects of smoking on treatment options, on the prognostic benefits of quitting and on reasons why some patients may continue to smoke [29]. Print materials may be preferable for patients with lower health literacy [29], and virtual education options (online assessments and emailed materials) have become essential during the COVID-19 pandemic [29]. Smart phone apps show promising effects in cancer clinic settings. An app specifically designed for cancer patients showed higher 2-month quit rates compared with a widely available general smoking cessation app when tested in a pilot randomised controlled trial [30]. Focussed training for health care workers and the appointment of “clinical champions” improved the implementation of a smoking cessation checklist in three Australian hospital oncology services, two metropolitan and one rural [31]. Systematic implementation of smoking cessation in cancer centres has not had extensive study and the Care to Quit Study, a stepped wedge cluster randomized, controlled trial (RCT), will investigate this across multiple sites in Australia [32]. Clinicians in cancer centres do need more training and may respond better to “micro interventions” such as short videos and brief courses than to comprehensive, multi-day courses that may be hard to balance with a busy clinical workload [29].

## 5. The Tobacco Endgame

Smoke-free tobacco endgame policies are emerging as a natural progression for countries which have had some success in tackling the smoking epidemic, surpassing the stipulations of the FCTC to attack both tobacco supply and demand. A tobacco endgame policy aims to move beyond controlling tobacco towards the phase-out or very restricted availability of tobacco. In the Asia-Pacific region, New Zealand has announced a commitment to ending the use of tobacco by 2025 with other countries including Australia developing policies that reach for more constrained goals.

New Zealand’s Smokefree Aotearoa 2025 plan [33] employs a comprehensive set of strategies attending to populations that have been particularly damaged by tobacco exposure. The plan itself has six focus areas: Māori leadership; funding health promotion and community activities; tailored “wrap-around” support; only having low-level nicotine tobacco products available (to reduce demand); fewer retail outlets (to reduce supply) and enforcing the law on tobacco industry and retailers. Close engagement with Māori leadership [34] has driven the plan, which represents a marked advocacy shift towards the elimination of tobacco (rather than containment) and towards censure of the industry rather than tolerance [35]. Considerable efforts over many years led to the development of this plan, with detailed attention to a range of policies at multiple government levels. Many recent outdoor smoking bans (dining, commercial areas, cultural events, campuses, sporting arenas) and support for tobacco-free retailers were introduced by local institutions and communities rather than by central government [36]. As far back as 2010, modelled data demonstrated that a smoke-free policy would particularly improve Māori life expectancy [37]. Early proposals included novel strategies such as direct reduction in retail supply of cigarettes and attracted strong public support [38]. The pressure of meeting a smoke-free goal of less than 5% by 2025, unlikely with “business as usual” [39] approaches to tobacco control, led to calls for the gathering of evidence through, for example, repeated cross-sectional surveys [40] and for new legislative strategies such as the focus on supply and much greater pressure on the tobacco industry [39]. The Smokefree Aotearoa 2025 plan attracted public support with most of the over 1000 respondents in each of two International Tobacco Control (ITC) surveys aware of it and over half supportive [41]. Qualitative research has investigated how people who smoke perceive the endgame goal [42]. Among 20 participants from marginalised communities, there were concerns that tobacco addiction was poorly understood, there was support for low-nicotine cigarettes, opposition to tax increases and that there was a tendency to use similar language to the tobacco industry in describing smoking as a choice. As New Zealand moves closer to its smoke-free goal, perceptions of smoking, denormalization of tobacco and greater support to help people quit will likely determine the success of the program.

Australia’s current National Tobacco Strategy 2012–2018 [43] prioritises nine issues that align with the principles of the FCTC. These include protection of public health policy, mass media campaigns, downward pressure on tobacco affordability, attention to the Aboriginal and Torres Strait Islander (ATSIC) community, attention to high-smoking communities, regulatory improvements, smoking bans and quit programs. At the time of writing, the Draft National Tobacco Strategy 2022–2030 [44] has undergone a consultation period before final release [45]. Additional priority areas include regulation to reduce supply of tobacco products, stronger regulation for novel and emerging products and broader smoking bans. The National Preventive Health Strategy 2021–2030 [46] aims for marked reductions in smoking rates in Australia by 2023 (to less than 5% generally and less than 27% in ATSIC communities), which echo the smoke-free goal in New Zealand. The planned strategies appear more conventional aiming to expand mass media campaigns, regulate content and product disclosures and to reduce supply through stronger regulation. Community partnerships would contribute to efforts in ATSIC peoples [46]. The tobacco control community in Australia has issued calls for stronger action including end dates for tobacco retail, based on consumer safety and ensuring support for retailers, with the overall aim of transitioning to a smoke-free society [47].

Endgame strategies are gaining traction internationally with several countries looking to take tobacco control further than stipulated by the FCTC. A review paper, looking at the far-reaching initiatives required to set firm tobacco-free target dates, has developed some “minimum requirements” that include public health consensus, public support, political champions to push through contentious legislation and collaboration across high and low-income communities to ensure equity [48]. Several countries so far have set endgame dates (Table 1) with aim to reduce smoking prevalence to 5% or less over the next 3 to 18 years [49]. Ireland aims to become tobacco free by 2025 through multiple initiatives including ‘denormalizing” tobacco use for upcoming generations [50]. The United Kingdom aims for less than 5% smoking rates by 2030 as part of personalised prevention efforts across a range of public health interventions [51]. The United States focuses on reductions in tobacco use in adults, increasing successful quit attempts and driving down tobacco product use in adolescents in its Healthy People 2030 objectives [52]. Sweden has developed tobacco control plans that endorse a smoke-free target by 2025 [53]. Finland introduced the endgame concept relatively early, in 2010, and recently brought the tobacco free aim closer, from 2040 to 2030 [54]. In 2013 in Scotland, the number of ex-smokers outnumbered current smokers for the first time; Scotland has since aimed for a tobacco-free generation by 2034, when children born in 2013 turn 21 years of age [55]. Canada aims to become tobacco free by 2035 with a range of measures that focus on support for quitting, protecting young people from nicotine addiction and working with indigenous groups to develop strategies specific to these communities [56]. According to its United Nations Sustainable Development Goals Report 2020, Bangladesh aims to become tobacco free by 2040 [57], but few details are available.

Key policies (Box 1) for the tobacco endgame may include those based on the product (low nicotine content, product standards to reduce appeal), on the user (licences or prescriptions, restriction by age/year of birth), on the market (ending commercial retail, reducing imports, markedly increasing tax), on regulatory straitjackets for the tobacco industry (such as fines for exceeding smoking prevalence targets) [49] and even criminal liability [58]. An interesting conceptual switch has been proposed, from prohibition (with the “spectres” of restriction and loss of choice) towards abolition (representing freedom) as a way to counter the rhetoric of the tobacco industry [59]. Growing international trends that expand smoking bans into the home, driven by Article 8 of the FCTC (Protection from exposure to tobacco smoke [60]), contribute to the denormalization of smoking and to smoke-free goals [61].

## 6. Tobacco Endgame and Cancer Centres

How would the tobacco endgame affect smoking cessation care in oncology? As of yet, no country has achieved lower than 5% smoking rates [62,63]. A social context with minimal smoking could contribute very positively to cancer care with potentially fewer new diagnoses of cancer, fewer cancer patients who actively smoke and an environment, through reduced demand and supply of tobacco products, that helps people quit. We could also anticipate positive effects on HCPs through further denormalization of smoking and, if done carefully, a reduction in stigma as smoking became viewed as a clinical condition needing treatment, rather than as a lifestyle factor. What are the risks? Could smoking cessation slip further down the priority list for HCPs? Would clinicians regard smoking as “dealt with”, and would those who continue to smoke come from vulnerable groups who already suffer poor outcomes and who would be less likely to engage with smoking cessation programs? As smoking rates fall, cancer clinics would need to develop an even sharper focus on equitable care to ensure that the benefits reached all people with cancer. How would tobacco endgame policies benefit patients who come from communities and countries with more permissive tobacco policies? Would endgame policies surprise them, would a very “anti-smoking” environment accentuate smoking-relating stigma and would these patients be reluctant to identify their smoking status and as a result miss out on care? Sensitive and supportive communications and treatment approaches will be required with particular care from HCPs to ensure that people with cancer who smoke have the best possible support for smoking cessation as part of cancer care.

## 7. Conclusions and Recommendations

Smoking cessation represents the fourth element in cancer treatment along with surgery, systemic therapy and radiation. Current evidence discussed in this paper confirms that smoking cessation remains overlooked in many cases, which is often due to clinician lack of confidence or training or even due to lack of time in busy cancer clinics. The impact of smoking cessation is clear; all evidence points to better outcomes in cancer patients who quit smoking, and a growing body of research is exploring better ways to bring effective smoking cessation into daily cancer care and to maximise the effects. Many countries and governments have started to focus on accelerating their efforts to reduce smoking rates, beyond the FCTC tenets of reduced demand, by dramatically reducing tobacco supply and by moving their societies towards tobacco free goals. This tobacco endgame, untested in its effects on cancer medicine, may change perceptions, advance clinical skills and ultimately improve lives for people with cancer, their families and the broader community.

Box 1 **Key points on the tobacco endgame for cancer care.**

Smoking cessation is underutilised in cancer clinics

Many cancer clinicians feel ill-equipped to provide effective smoking cessation therapy

Effective quitting leads to much better cancer outcomes

Tobacco control is moving from addressing demand to radically reducing supply

The tobacco endgame aims to reduce smoking rates below 5%

The impact of the tobacco endgame is untested in cancer medicine but has the potential to change the therapeutic environment

## Figures and Tables

**Table 1:** Current international tobacco endgame goals [49].

Country	Target Smoking Rate	Date
New Zealand	<5%	2025
Ireland	<5%	2025
Sweden	<5%	2025
United Kingdom	Smoke-free	2030
USA	<5%	2030
Australia	<5%	2030
Finland	<5%	2030
Scotland	<5%	2034
Canada	<5%	2035
Bangladesh	Tobacco free	2040
